# Digital Age and Medicine: Visualization and Evaluation of Foot Anatomy with Artificial Intelligence

**DOI:** 10.3390/diagnostics15050550

**Published:** 2025-02-25

**Authors:** Ferda Başgün, Tuba Altunbey, Sevinç Ay, Derya Öztürk Söylemez, Elif Emre, Nurseda Başgün

**Affiliations:** 1 Vocational School of Technical Sciences, Fırat University, Elazığ 23000, Turkey; 2 Public Relations and Publicity, Fırat University, Elazığ 23000, Turkey; tcanak@firat.edu.tr; 3 Department of Software Engineering, Fırat University, Elazığ 23000, Turkey; say@firat.edu.tr; 4 Vocational School of Health Services, Sinop University, Sinop 57000, Turkey; deryaozturk@sinop.edu.tr; 5 Faculty of Medicine, Department of Anatomy, Fırat University, Elazığ 23000, Turkey; eemre@firat.edu.tr; 6 Physiotherapy and Rehabilitation, Fırat University, Elazığ 23000, Turkey; nursedabasgun@gmail.com

**Keywords:** medical illustration, foot anatomy, artificial intelligence

## Abstract

**Background/Objectives**: Artificial intelligence (AI) has attracted great interest due to its applicability in many fields. The adoption of visual illustration techniques produced by AI in the field of graphic design has further expanded the field of use of this technology. This study focuses on foot anatomy illustrations generated by Adobe Firefly and Microsoft Designer Image Creator applications, evaluating them based on detail, clarity, anatomical realism, accuracy, and aesthetic appeal. **Methods**: The illustrations were created using text-based scripts, and five anatomists compared them to traditional illustrations from the Sobotta Atlas of Human Anatomy. **Results**: Fleiss’ Kappa statistic was used to analyze consistency among expert evaluations. For the four figures generated by both AI applications, Fleiss’ Kappa agreement was high. Adobe Firefly performed slightly better in illustrating phalanx and ankle bones, but its anatomical accuracy was lower for tarsal and metatarsal bones. Microsoft Designer Image Creator excelled in illustrating metatarsal bones, while its tarsal and phalanx illustrations were less anatomically accurate than Adobe Firefly and the atlas drawings. Both programs showed average realism in ankle structures, while the tarsal bones had low realism. **Conclusions**: Artificial intelligence applications within the scope of the study showed fast performance. Aesthetic appeal is dominant at first glance in the resulting drawings. In general, both applications have struggled to reflect anatomical reality.

## 1. Introduction

The employment of anatomy-based AI applications in orthopedic surgery and imaging contributes to the potential to enhance clinical decision-making and optimize patient care [1,2,3]. Furthermore, the advent of sophisticated artificial intelligence technologies has engendered new opportunities for the production of high-quality, precise images in the field of anatomy [4]. In addition, medical illustrations can be used to visualize the human body more clearly and understandably in scientific research and social information, especially in medical education [5]. AI-assisted image generators have the ability to produce a significant amount of concept art in a short period of time. Unlike human illustrators, who have to work for days to make revisions, AI can deliver a completely different set of color images in minutes. This rapid illustration creation allows different concepts and ideas to be explored quickly, giving educators a wide variety of visual examples to choose from [6]. This study involves a comparative analysis of orthopedics-based foot anatomy images prepared through artificial intelligence applications and an evaluation of the results by experts.

## 2. Importance of Medical Illustration in Healthcare

Medical illustrations are an increasingly significant field in health knowledge transfer and the promotion of health literacy [7]. These visual representations play a crucial role in the comprehension of complex medical information, facilitating understanding of textual information in the field of medicine through their incorporation into materials such as brochures and textbooks. Furthermore, medical illustrations offer a visualization of complex images and body parts in the medical field [8]. In the field of medical illustration, user requirements have been identified as a crucial factor in optimizing the effectiveness of these visual aids. A study conducted at Auckland City Hospital revealed that patients generally exhibit a preference for illustrations that provide a greater volume of information, while medical professionals expressed satisfaction with illustrations that are less exhaustive [9]. The significance of medical illustrations extends beyond the realm of education, encompassing crucial domains such as public health and preventive medicine [10]. Furthermore, the importance of medical illustrations at different levels of medical education has increased, especially with the development of new technologies and teaching methods [11]. Medical illustrations are distinguished from other types of illustrations by virtue of the complex anatomy of human beings [12]. Consequently, the designer who will create medical illustrations scientifically must be experienced in their profession and collaborate with an expert who possesses technical knowledge about the structure to be illustrated. In the contemporary field of anatomy education, the use of medical illustrations that create text and visuals supported by artificial intelligence is important. However, to ensure the accuracy and realism of these technologies, it is essential to augment training databases with anatomically accurate images [6]. These developments underscore the pivotal role of medical illustrations in healthcare and the imperative for novel and innovative approaches in this field.

## 3. Orthopaedic Medical Illustrations with Artificial Intelligence Applications

In recent years, significant advancements have been made in the field of medicine thanks to the development of artificial intelligence technologies. These technologies have started to offer remarkable suggestions in specific areas of medicine, such as orthopedic imaging. The use of orthopedic images in medical illustration has led to revolutionary innovations in the processes of image analysis and image creation [13]. One of the most prominent artificial intelligence applications in the field of orthopedic imaging is the analysis of radiographs. The utilization of deep learning algorithms has demonstrated notable advancements in domains such as fracture detection, osteoarthritis classification, and bone age assessment. These algorithms have been shown to perform comparably to or even superior to human readers, yet current standards require the confirmation of expert human validation [14,15]. In the field of orthopedic surgery, there has been a recent emergence of artificial intelligence-based illustration images, which have found application in the analysis of radiographs following total hip and knee arthroplasty. This development represents a significant advancement in the realm of orthopedic AI research. The potential of AI-based image analysis lies in its ability to automate processes such as implant position verification and complication identification. These applications have been shown to offer high accuracy in implant identification and the prediction of implant failure risk [16]. As asserted by Cofaru et al. [17], detailed illustrations of the foot’s intricate anatomy can facilitate a comprehensive understanding of the complex structures within the foot for orthopedic surgeons. Illustrations of this nature, often derived from cadaveric dissections, have been demonstrated to assist surgeons in visualizing the precise location and relationships of bones, muscles, tendons, and ligaments [18]. This visualization is imperative for ensuring safe and effective surgical interventions. Furthermore, an additional application of artificial intelligence in orthopedic surgery is the study of social perceptions through text-based image generation systems. These systems offer the opportunity to explore the perceptions of orthopedic surgeons in society by creating images representing gender and racial diversity. However, the diversity in the images generated by such systems can differ significantly when compared to real-world data [19]. The human foot is a complex structure formed by many bones, joints, and muscles. Its primary functions include transferring body weight to the ground, facilitating movement, and providing feedback on proprioceptive sensations [20]. The comprehension of the complex anatomy of the foot is of significant importance to clinicians, orthopaedists, and researchers studying gait mechanics, as well as to those developing footwear and orthotic devices [17]. While the potential of AI programs in the field of orthopedic imaging is considerable, further research is required to facilitate the widespread integration of these technologies into clinical applications. The development of robust studies designed in accordance with standard reporting guidelines is encouraged to facilitate the easy adaptation of AI models to real-world conditions [21,22]. Consequently, it is evident that artificial intelligence technologies offer significant innovations in the field of orthopedic imaging, with a growing variety of applications in this domain. However, further research and development is required for these technologies to be widely adopted in clinical applications. Additionally, the accuracy of orthopedic images produced by artificial intelligence applications should be evaluated by experts and medical illustrations should be incorporated into artificial intelligence programs to facilitate the analysis of results.

## 4. Materials and Methods

**Figure 1:**
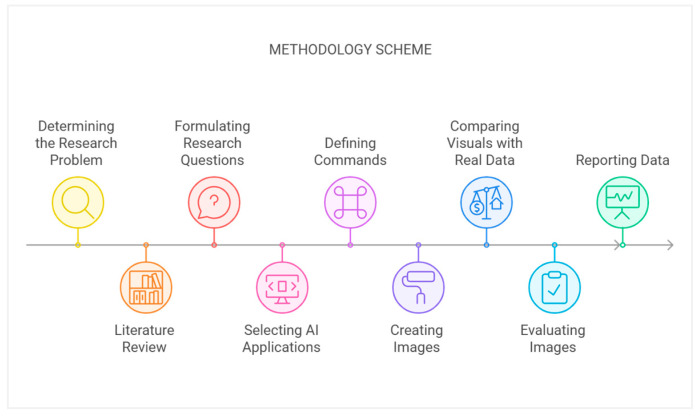
The methodology scheme.

This study aims to create orthopedic illustrations in artificial intelligence programs and evaluate these medical illustrations in terms of detail, clarity, anatomical realism and accuracy, lack of imaginary anatomy and aesthetic appeal. The method scheme created for this purpose is as follows (see Figure 1):

This research evaluates the realism of medical illustrations generated by artificial intelligence programs for foot anatomy. To achieve this, a literature review was conducted based on specific criteria.

Two artificial intelligence programs that can produce medical illustrations (Adobe Firefly and Microsoft Designer Image Creator) were selected for this study. These programs were preferred because they allow users to experiment with many different designs in a short time, produce fast and detailed content, and offer a user-friendly interface and easy access. The selected artificial intelligence applications were given commands determined by experts in the field of anatomy. In line with the commands given, Adobe Firefly and Microsoft Designer Image Creator applications offered more options in terms of anatomy suitability and diversity. In this context, these two AI applications stood out and were selected due to their level of sophistication and impressive performance in visual drawings.

Adobe Firefly, developed by Adobe as a content creation platform, utilizes Adobe’s artificial intelligence technologies. Notably prominent in the field of generative artificial intelligence (Generative AI), Firefly, introduced in 2023, aims to streamline original design processes and enable users to bring their creative ideas to life more efficiently and effectively [23]. While Adobe Firefly contributes to various disciplines, it has proven particularly innovative and effective in the creation of medical illustrations. Microsoft Designer Image Creator is the other artificial intelligence application program selected for this study. This Microsoft-based program uses generative artificial intelligence technologies such as Adobe Firefly to create high-quality images from text-based input [24]. This platform integrates OpenAI’s DALL-E model [25]. Preliminary sketching studies in two selected artificial intelligence drawing programs have shown that these programs recognize the word medical illustration. In this context, it has been experienced that artificial intelligence applications create medical illustration drawings with commands for the medical field. Given the complexity and importance of foot anatomy in the human body, this study was selected as “visualization and evaluation of foot anatomy with artificial intelligence”. The artificial intelligence programs to be used within the scope of this study were given commands expressed by different word selections with 20 similar content chosen by experts in the field of anatomy. The commands selected for ankle anatomy include 7 tarsal bones, 5 metatarsal bones, 14 phalangeal bones, the ankle joint, and the anatomical aspects that make up these structures. The focus was on visualizing the entire foot and ankle joint, including specific bones and joint relationships, to provide detailed anatomical context with the given commands. With the given commands, the AI did not fully understand the medical and anatomical terms. Therefore, while the visualizations were aesthetically pleasing, they lacked anatomical accuracy. Experts noted that the visualizations created based on the detailed terms in the commands were more complex and difficult to understand. Based on expert feedback emphasizing simplicity and comprehensibility, the command “create anatomically correct medical illustration drawing by detailing the foot bones to show the foot and ankle joint” was selected for this study. The medical illustrations generated through this command, as well as reference images from the Sobotta Atlas of Human Anatomy, are presented in the findings section. Academics and professionals with clinical experience and expertise in the field of anatomy and medical illustration evaluated the illustrations created by artificial intelligence programs in the categories of detail, clarity, aesthetic appeal, lack of fictitious anatomy, anatomical realism, and accuracy [3].

**Figure 2:**
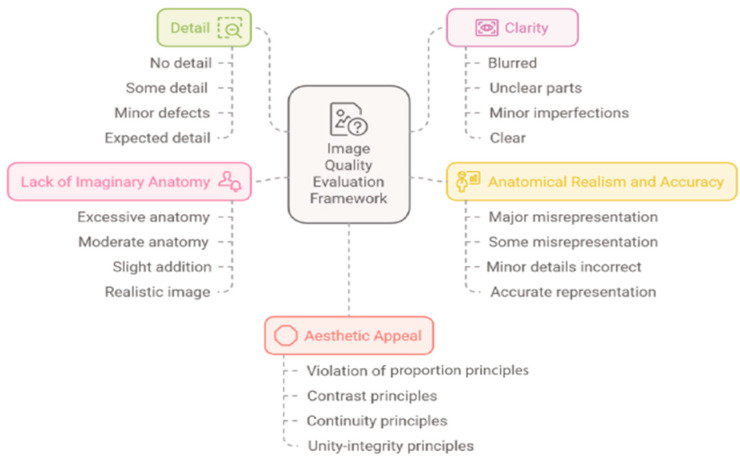
Scoring criteria.

Each category was scored on a scale from 0 to 3 [3]. The scheme for scoring criteria is given in Figure 2. In the scoring criteria, the anatomical reality of the foot region was evaluated by taking into account the ankle joint structure, tarsal bones, metatarsal bones, phalanx bones, muscle structures, nerve, and vascular structures. When compared with the reference human anatomy atlas images, 3 points were given if all major and minor anatomical structures were represented correctly, 2 points were given if major anatomical structures were correctly represented and minor anatomical structures were incorrectly represented, 1 point was given if some of the major anatomical structures were correctly represented and minor anatomical structures were incorrectly represented, and 0 points were given if there was a discrepancy and inaccuracy in all anatomical structures. In the evaluation of anatomical images in terms of aesthetic appeal, the principles of unity and integrity, continuity, contrast, and ratio-proportion were established based on graphic design principles. According to the principle of unity and integrity, if the image is harmonious and consistent in terms of color, drawing, and subject matter, 3 points were awarded; according to the principle of continuity, drawings that continue the eye movement were awarded 2 points; regarding the principle of contrast, colors (light and dark relationship) were awarded 1 point, and images created in violation of the ratio-proportion principle were awarded 0 points. When evaluating the category of lack of fictitious anatomy, images with excessive additional fictitious findings received 0 points; images with moderate additional anatomical findings received 1 point; images with a slight additional anatomy that does not detract from the overall image received 2 points; and images presented realistically without a fictitious anatomy received 3 points. In the clarity category, 0 points were awarded if the image was blurred and unclear; 1 point if important parts of the image were unclear; 2 points if the image was clear with minor imperfections; and 3 points if the image was clear and understandable as expected. The criteria for scoring the detail category are as follows: 0 points if the image lacks detail and is vague; 1 point if the image has some detail; 2 points if the image is detailed with minor imperfections; and 3 points if the image is detailed as expected. Classification categories for scoring criteria are shown in Figure 2.

## 5. Results

The foot anatomy illustrations generated by the commands given to the artificial intelligence applications and the images of the illustrations in the Sobotta Atlas of Human Anatomy are shown in Table 1. The four-foot anatomy images in Table 1 were evaluated by five experts. The evaluation results for each image are summarized and explained using graphical representations.

In Table 1, the command ‘create anatomically correct medical illustration drawing by detailing the foot bones to show the foot and ankle joint’ was given to the selected artificial intelligence application programs. Each application generated four images simultaneously in response to this single command. Expert opinions on the images created are given below.

In the images created by the Microsoft Designer Image Creator application, the distal parts of the os tibia and os fibula that make up the ankle joint look realistic, but the os talus does not reflect reality in terms of shape. The os talus is a larger bone that should articulate with the os tibia and os fibula. The os calcaneus bone under the os talus is depicted in accordance with its real shape and size. While there should be five more tarsal bones other than os talus and os calcaneus (os naviculare, os cuneiforme mediale, os cuneiforme intermedium, os cuneiforme laterale, os cuboideum), 12 tarsal bones are pictured in the image, and their shapes and alignments are not correct. Although five metatarsal bones are close to real in number and shape, their lengths are drawn incorrectly. Phalanx bones should be two on the first finger and three on the other fingers. Although the bone structure cannot be seen completely due to the nail and skin details on the fingertips, it is noteworthy that the number of bones is high, especially on the second finger. Muscles, ligaments, and tendons on the dorsal part of the foot are not depicted in the present image. Only the mm. interossei dorsales pedis muscles between the metatarsals are visible. Other muscle drawings added to the image could not be matched anatomically with any muscle. There are no main artery and vein formations in the foot region, and the vascular structures depicted cannot be named. Although it is a visual that gives an idea about the anatomy of the foot, it is not suitable for learning anatomy. In terms of anatomical reality, it can be said that some prominent anatomical structures are misrepresented.

In the images created by Adobe Firefly, the distal parts of the os tibia and os fibula that make up the ankle joint look realistic, while the os talus is drawn completely wrong. os calcaneus bone is not clear because it is covered with tissues. All of the tarsal bones are far from the real shape and number. Although the metatarsal bones resemble the real ones in length, they were evaluated with low success in terms of shape, number, and clarity. Phalanx bones should be two on the first finger and three on the other fingers. Since there are nail and skin details on the fingertips, the bone structure cannot be seen completely, but it is considered to be close to realistic in length. Muscle, vessel, and nerve formations are not depicted in the drawing. Although there are a few ligament-like drawings, they are not clear enough to be named.

**Figure 3:**
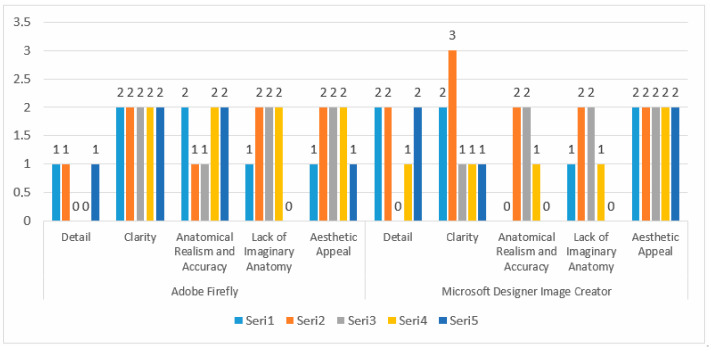
Comparison and evaluation of AI applications related to Figure 3.

The drawings of the ankle structures of both programs have an average level of realism, while the tarsal bones have a low level of realism and are not superior to each other. When all structures are evaluated together, both programs are weak in terms of reflecting anatomical reality. The scoring of each image evaluated by the experts and the images generated by the AI applications are presented in the order in which they appear in Figure 3.

**Figure 4:**
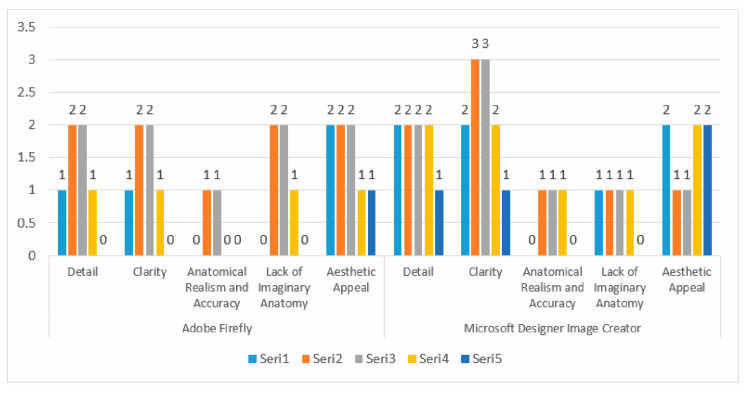
Comparison and evaluation of AI applications related to Figure 4.

The graph presents a comparison of two image creation tools, Adobe Firefly, and Microsoft Designer Image Creator, based on specific attributes. The analyzed attributes include detail, clarity, anatomical realism, accuracy, lack of imaginative anatomy, and aesthetic appeal. Adobe Firely received the highest score of 1 in the ‘Detail’ category, and its illustrations are vague and lack detail. In contrast, Microsoft Designer Image Creator received the highest score of 2 in the ‘Detail’ category. Microsoft Designer Image Creator performed better than Adobe Firely in the ‘detail’ category. Adobe Firely received two points from all experts in the ‘Clarity’ category. Microsoft Designer Image Creator received the highest score of 3 and the lowest score of 1 in the ‘Clarity’ criterion. Adobe Firefly and Microsoft Designer Image Creator, both tools, received a maximum score of 2 in the ‘Anatomical Realism and Accuracy’ category. Experts note that illustrations created by Adobe Firefly demonstrate greater anatomical accuracy compared to those produced by Microsoft Designer Image Creator. Under the Lack of Imaginary Anatomy category, both applications scored 2 points. Experts consider the ability of these tools to create imaginary anatomies to be a slight addition that does not detract from the overall image. In the Aesthetic Appeal category, both applications received an average score of 2. This indicates that both tools have a satisfactory level of aesthetic appeal but are not perfect. The results are also shown in Figure 4 with numerical values.

**Figure 5:**
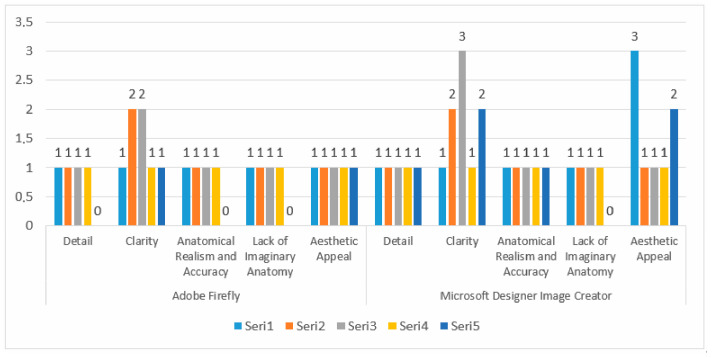
Comparison and evaluation of AI applications related to Figure 5.

Both Adobe Firefly and Microsoft Designer Image Creator scored 2 in the ‘Detail’ category, indicating that both tools perform similarly in terms of detailing. Experts suggest that neither system provides sufficient detail. In the ‘Clarity’ criterion, Microsoft Designer Image Creator demonstrates a clear superiority with a score of 3, while Adobe Firefly scored only 2. This finding can be interpreted as Microsoft being more successful in terms of clarity. Both applications scored 1 in the ‘Anatomical Realism and Accuracy’ criterion, indicating that both applications misrepresent in terms of anatomical accuracy. Experts note that both tools fail to meet expectations in terms of anatomical realism. Adobe Firefly received the highest score of 2 in the ‘Lack of Imaginary Anatomy’ category, while Microsoft Designer Image Creator scored 1. This indicates that Microsoft Designer Image Creator introduces more extreme, imaginary anatomical features compared to Adobe Firefly. In terms of aesthetic appeal, both applications performed equally. The performance values of the programs are given in Figure 5.

**Figure 6:**
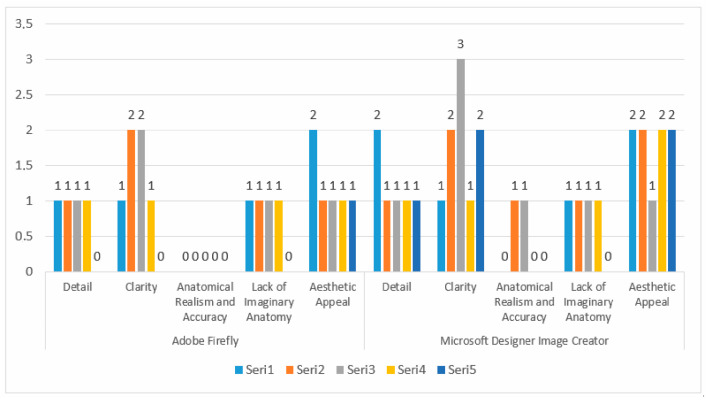
Comparison and evaluation of AI applications related to Figure 6.

Adobe Firefly and Microsoft Designer Image Creator scored 1 in the ‘Detail’ criterion. This shows that both applications are ambiguous in terms of detail. Microsoft Designer Image Creator holds a clear advantage in the ‘Clarity’ category with a score of 3, while Adobe Firefly scored 2. This suggests that Microsoft provides better results in terms of clarity. Both applications scored 1 in the ‘Anatomical Realism and Accuracy’ criterion, indicating that both have deficiencies in anatomical realism and accuracy in the ‘Lack of Imaginary Anatomy’ criterion. The average of both applications is equal to each other. This shows that both programs have a moderate level of drawing imaginary anatomical formations that should not be in the visual. In terms of ‘Aesthetic Appeal’, Microsoft Designer Image Creator received the highest score of 3, while Adobe Firefly received 1. This result indicates that Microsoft performs better in terms of aesthetic appeal. The results obtained are given in Figure 6.

An expert evaluation of both applications based on the ‘Detail’ criterion reveals that the average score is 1, indicating that both Microsoft Designer Image Creator and Adobe Firefly produce illustrations lacking in detail. In the ‘Clarity’ criterion, Microsoft Designer Image Creator scored 3 points, showing a slight superiority over Adobe Firefly, which scored 2 points. Both Adobe Firefly and Microsoft Designer Image Creator produce illustrations that exhibit misrepresentations and errors in terms of ‘Anatomical Realism and Accuracy’. In the ‘Lack of Imaginary Anatomy’ category, the performance of both applications is identical, with experts agreeing on this aspect. Experts agree in this area. In the ‘Aesthetic Appeal’ criterion, Microsoft Designer Image Creator performed better than Adobe Firefly. Microsoft Designer Image Creator scored 2 points, while Adobe Firefly scored 1 point. This result reveals that Microsoft performs better in terms of aesthetics.

### Human Assessment Analysis

Medical illustration drawings generated by two different artificial intelligence applications were scored between 0 and 3 by experts. Fleiss’s Kappa statistic was used to assess how well the experts agreed about the medical illustration drawings. Fleiss Kappa is widely used, particularly in medicine, psychology, and social sciences, to measure the agreement among multiple raters. Fleiss’ Kappa is a statistical method used to measure the consistency (inter-rater agreement) of categorical decisions made by three or more raters. While Cohen’s Kappa only works for two raters, Fleiss’ Kappa is used in situations involving multiple raters [26]. Within the scope of this study, calculations were performed using the ‘irr’ or ‘psych’ packages in the R program. The formula used in the calculation of Fleiss Kappa is as follows:

These values help assess how well multiple raters agree on categorical classifications, adjusting for chance agreement [26].

(1) $$\kappa =\frac{\bar{P}-\bar{P_{e}}}{1-\bar{P_{e}}}$$

$\bar{P}$ (Observed agreement): It is the rate of agreement between the true observations. $\bar{P_{e}}$ (expected agreement): It describes how the observed, true fit is beyond randomness. κ is calculated by subtracting the expected agreement from the observed agreement between raters [27].

$\bar{P}$ = Observed agreement, $\bar{P_{e}}$ = Expected agreement by chance, Observed Agreement $\left(\bar{P}\right)$ Calculation

(2) $$\bar{P}=\frac{1}{N}\sum_{i=1}^{N}P_{i}$$

N = Total number of subjects (items being rated), $P_{i}$ = Agreement proportion for subject

(3) $$P_{i}=\frac{1}{k(k-1)}\sum_{j=0}^{3}n_{j\;}[n_{j\;}-1]$$

K = 5 (Number of experts).

The interpretation table for Fleiss’ Kappa values, commonly used in determining interrater reliability, is as follows in Table 2:

This table is used to understand the degree of agreement among raters. A higher Kappa value indicates a greater level of agreement.

The tables obtained from the expert evaluation of medical drawings obtained from two different artificial intelligence applications are given below. In the tables, Figure 1, Figure 2, Figure 3 and Figure 4 show the item-specific agreement ${\bar{P}}_{i}$ values of five criteria (Detail, Clarity, Anatomical Realism, Lack of Image Anatomy, and Aesthetic Appeal). In the last column, the Fleiss’ Kappa (General) for the 5 items in Table 3 is presented.

In Figure 1 (Adobe Firefly), the ${\bar{P}}_{i}$ values for the criteria are distributed as (0.40, 1.00, 0.30, 0.40, 1.00), and their Fleiss’ Kappa is calculated as 0.28 (fair to moderate agreement). Figure 2 showed high agreement (0.81. Since all criteria were scored almost the same in Figure 3 and Figure 4, each item is shown in the table as ${\bar{P}}_{i}$ = 1.00 and Fleiss’ Kappa as 1.00 (perfect agreement). This indicates that the evaluations are not entirely random but still contain a certain level of subjective differences. Microsoft Designer Image Creator Expert Evaluation Results are presented in Table 4.

According to the table in Figure 1 (Designer), the item-specific ${\bar{P}}_{i}$ values for the criteria vary (1.00, 0.30, 0.40, 1.00, 0.60), which results in an overall Kappa of 0.52 (moderate agreement). Figure 2 and Figure 4 have higher agreement (0.85 and 0.87). Although Figure 3 gives an average item fit of 0.84, Fleiss Kappa was calculated as 0.70, according to the expected fit.

As a result of the Fleiss’ Kappa analyses, the differences in rating behaviors reveal the subjective variability in the evaluations. Additionally, it is observed that generally consistent evaluations were obtained among the reviewers.

## 6. Discussion

Artificial intelligence technologies are contributing more and more to the field of medicine as they address many fields of science. This technology has the potential to contribute to specialized fields such as orthopedic surgery and imaging, as well as many other areas of medicine. The potential of AI applications in medical fields such as orthopedic surgery is extremely important in terms of improving the accuracy of medical processes, enabling faster and more effective decisions for treatment, and making surgical processes safer. However, for AI to fully realize this potential, improvements in training data sets are required. Diversifying the data, improving their quality, and labeling them correctly will enable AI to produce more reliable and accurate results and overcome limitations in the medical field. AI-enabled systems can offer a wide range of innovations, from medical illustrations to radiographic analysis. Recent studies have even shown significant improvements in clinical decision-making and patient care. While AI technologies have achieved widespread success in recognizing data sets or diagnosing disease, the lack of anatomical realism and difficulties in accurately depicting complex structures, such as bones and joints, remain obstacles to overcome in clinical practice. The latest innovations in artificial intelligence technologies are developing through image generation software that draws commands given in text. Medical illustration, which is drawn by specific experts in the field of graphic design, is discussed in this article with the support of artificial intelligence. This article aims to discuss the role of AI-based medical illustrations, especially in the field of orthopedic imaging. Medical illustrations have an important place in the medical field, and they have the potential to make complex anatomy and physiology information more understandable. These illustrations, which are carefully created with traditional methods, can be produced quickly and flexibly with the development of artificial intelligence-assisted technologies in recent years. One of the main advantages of AI-assisted illustrations is that they offer fast production processes. A design process that takes weeks in traditional illustrations can be created in minutes with these technologies. However, expert validation and further research are required for these applications to become widespread in clinical practice. The development of AI-assisted illustration and imaging technologies is likely to face different challenges in the medical field. In particular, the objectivity and adequacy of the data sets used during the learning process of AI models will play a decisive role in the anatomical accuracy of these technologies. In the AI-supported foot anatomy drawings made within the scope of this study, it was concluded by the experts that the AI drawings did not reflect anatomical accuracy. The main reasons for this situation can be interpreted in several different ways. First, the limitations of AI in accurately drawing human anatomy are directly proportional to the inherent complexity of the human form. Another factor is the difficulties in representing anatomical nuance, simulating the depth and three-dimensional nature of the body [28,29], and the fact that AI models rely heavily on internet data in their training process. The lack of accurate anatomical images or the prevalence of misinformation on the Internet are important limitations. In this context, it causes the AI to have difficulty distinguishing between true and false and, therefore, to produce erroneous outputs by learning incorrect or incomplete information. In order to overcome this problem, it is of great importance to improve the quality of training data sets, feed them from accurate and reliable sources, and provide data diversity. In addition, auditing and verification of the outputs of AI models by human experts can significantly contribute to minimizing such errors. While AI has made notable strides in several areas, the unique challenges posed by human anatomy suggest that there is still significant room for improvement before AI can match the accuracy and artistry of skilled human anatomical illustrators.

Although the two applications selected in the study gave four drawings with a single command, a fully accurate medical illustration could not be created from these drawings. Although these applications attract attention with their aesthetic appeal, it is necessary to emphasize that these applications provide more accurate results. Collaboration between medical illustrators and artificial intelligence developers to create more anatomically accurate images may be recommended. In this context, new and accurate visual data sets should be created in which field experts and medical illustration artists work together. These data sets will be taught to artificial intelligence software and will provide more accurate results. This contribution to the field of anatomy will be a guide that can be adapted to other fields of medicine as a guide.

## 7. Conclusions

The Fleiss Kappa agreement between experts in images created using Adobe Firefly (Generative AI 3) and Microsoft Designer Image Creator (OpenAI’s DALL-E model) artificial intellegence programs has generally shown consistent results. Although both AI programs have certain strengths in terms of aesthetic appeal and rapid image generation, they have not fully achieved anatomical realism and accuracy. Significant improvements in anatomical accuracy are necessary to utilize AI programs in medical applications. Future studies should focus on developing AI technologies through interdisciplinary collaborations to achieve higher realism and accuracy.

## Figures and Tables

**Table 1:** Anatomical illustrations were created using artificial intelligence applications and Sobotta Human Anatomy Atlas illustrations.

Adobe Firefly	Microsoft Designer Image Creator	Anatomy Atlas Image
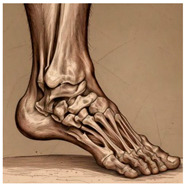	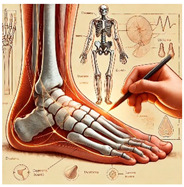	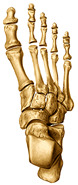
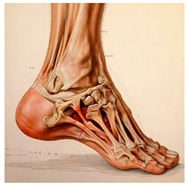	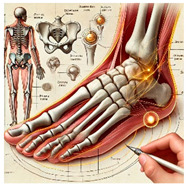	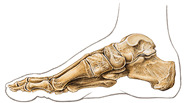
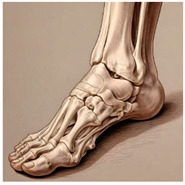	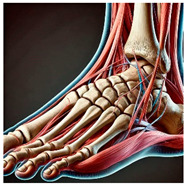	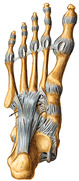
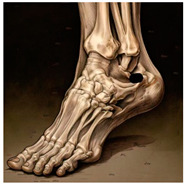	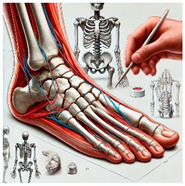	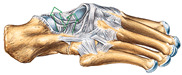

**Table 2:** Fleiss’ Kappa values.

Kappa Value (*κ*)	Level of Agreement
<0	Poor agreement (Disagreement)
0−0.20	Slight agreement
0.21−0.40	Fair agreement
0.41−0.60	Moderate agreement
0.61−0.80	Substantial agreement
0.81−1.00	Almost perfect agreement

**Table 3:** Adobe Firefly expert evaluation result.

Figure	Detail (${\bar{P}}_{i}$)	Clarity (${\bar{P}}_{i}$)	Anatomical (${\bar{P}}_{i}$)	Lack of Image (${\bar{P}}_{i}$)	Aesthetic (${\bar{P}}_{i}$)	Fleiss’ Kappa Score
Figure 1	0.40	1.00	0.30	0.40	1.00	0.28
Figure 2	0.40	1.00	1.00	1.00	1.00	0.81
Figure 3	1.00	1.00	1.00	1.00	1.00	1.00
Figure 4	1.00	1.00	1.00	1.00	1.00	1.00

**Table 4:** Microsoft Designer Image Creator Expert evaluation results.

Figure	Detail (${\bar{P}}_{i}$)	Clarity (${\bar{P}}_{i}$)	Anatomical (${\bar{P}}_{i}$)	Lack of Image (${\bar{P}}_{i}$)	Aesthetic (${\bar{P}}_{i}$)	Fleiss Kappa Score
Figure 1	1.00	0.30	0.40	1.00	0.60	0.52
Figure 2	1.00	0.40	1.00	1.00	1.00	0.85
Figure 3	1.00	0.60	1.00	1.00	0.60	0.70
Figure 4	1.00	0.60	1.00	1.00	1.00	0.87

## Data Availability

The raw data supporting the conclusions of this article will be made available by the authors on request.
